# A new corrosion-inhibiting strategy for biodegradable magnesium: reduced nicotinamide adenine dinucleotide (NADH)

**DOI:** 10.1038/s41598-018-36240-3

**Published:** 2018-12-10

**Authors:** Jimin Park, Minjung Park, Hyunseon Seo, Hyung-Seop Han, Ji-Young Lee, Dongkyu Koo, Kyeongsoo Kim, Pil-Ryung Cha, James Edwards, Young-Woon Kim, Kang-Sik Lee, Myoung-Ryul Ok, Hojeong Jeon, Hyun-Kwang Seok, Yu-Chan Kim

**Affiliations:** 1 Center for Biomaterials, Korea Institute of Science and Technology (KIST), Seoul, 02792 Republic of Korea; 2 Department of Materials Science and Engineering, Massachusetts Institute of Technology, Cambridge, Massachusetts, 02139 USA; 3 Department of Materials Science and Engineering, Seoul National University, Seoul, 08826 Republic of Korea; 4 Nuffield Department of Orthopaedics, Rheumatology and Musculoskeletal Sciences, University of Oxford, Oxford, OX37LD UK; 5 School of Advanced Materials Engineering, Kookmin University, Seoul, 02707 Republic of Korea; 6 Biomedical Engineering Research Center, Asan Institute for Life Sciences, Asan Medical Center, College of Medicine, University of Ulsan, Seoul, 05505 Republic of Korea; 7 Division of Bio-Medical Science and Technology, KIST School, Korea University of Science and Technology, Seoul, 02792 Republic of Korea

## Abstract

Utilization of biodegradable metals in biomedical fields is emerging because it avoids high-risk and uneconomic secondary surgeries for removing implantable devices. Mg and its alloys are considered optimum materials for biodegradable implantable devices because of their high biocompatibility; however, their excessive and uncontrollable biodegradation is a difficult challenge to overcome. Here, we present a novel method of inhibiting Mg biodegradation by utilizing reduced nicotinamide adenine dinucleotide (NADH), an endogenous cofactor present in all living cells. Incorporating NADH significantly increases Mg corrosion resistance by promoting the formation of thick and dense protective layers. The unique mechanism by which NADH enables corrosion inhibition was discovered by combined microscopic and spectroscopic analyses. NADH is initially self-adsorbed onto the surface of Mg oxide layers, preventing Cl^−^ ions from dissolving Mg oxides, and later recruits Ca^2+^ ions to form stable Ca-P protective layers. Furthermore, stability of NADH as a corrosion inhibitor of Mg under physiological conditions were confirmed using cell tests. Moreover, excellent cell adhesion and viability to Mg treated with NADH shows the feasibility of introduction of NADH to Mg-based implantable system. Our strategy using NADH suggests an interesting new way of delaying the degradation of Mg and demonstrates potential roles for biomolecules in the engineering the biodegradability of metals.

## Introduction

Biodegradable metals have drawn extensive attention as core materials for biomedical applications due to their biodegradability and compatible mechanical and electrical properties for human body^1–5^. A myriad of implantable devices were developed utilizing biodegradable metals and these devices are naturally absorbed by the human body after finishing their biomedical missions such as tissue replacement^1^, vascular intervention^2^, drug delivery^3^, and the monitoring or stimulation of biosignals^4,5^. Among several biodegradable metal candidates, Mg has received a big spotlight in the global market owing to its high absorbability and appropriate mechanical properties matching with tissues^6–8^. Furthermore, studies regarding biological interactions between cells and Mg ions have revealed that Mg ions positively affect various cells and their functions^9–12^. For example, Zhu *et al*. showed that Mg enhanced osteogenic differentiation of human bone marrow mesenchymal stem cells, which can be followed by bone regeneration required for orthopedic implants^12^. Therefore, numerous studies demonstrated the effectiveness of Mg-based implants in clinical settings^13–16^.

Unfortunately, the utilization of Mg for implantable devices is a constant concern due to its intrinsically high biodegradation rate. Mg exhibits poor corrosion resistance in biological fluids containing large concentrations of ions^17^. The excessive corrosion of Mg-based implants *in vivo* results in device failure, causing a loss of performance before they complete their biomedical purpose. Furthermore, hydrogen gas produced from Mg corrosion causes pressure-induced stress on adjacent tissues and interfacial failure between Mg and tissues, deteriorating its biocompatibility^8,18,19^.

Therefore, numerous efforts were made to improve the corrosion resistance of Mg. For example, using an appropriate alloy design strategy effectively decreases Mg corrosion rates^20–24^. Our previous reports demonstrated that Mg degradation is significantly reduced with alloying elements by synchronizing the corrosion potentials of two constituent phases^25^. A clinical trial showed that a proposed Mg alloy (Mg-5wt%Ca-1wt%Zn) degraded slowly during the bone healing process^23^. In addition, Witte *et al*. successfully decreased *in vivo* degradation rates of Mg alloys using Al, Zn, and other rare earth elements as additives^24^. However, the addition of new elements to Mg carries potential risks as these elements might not be biocompatible and could produce unexpected side effects.

As an alternative method, coating materials have been suggested to prevent metals from reacting with corrosive biological environments^25–28^. Recently, Zang *et al*. successfully fabricated Cu-thiolate-coated TiO_2_ superhydrophobic layers to increase the corrosion resistance of Mg alloys^25^. Moreover, various polymer and ceramic materials such as poly-L-lactide (PLLA)^26^, polycaprolactone^27^, and hydroxyapatite^28^ have been applied as coating layers on Mg surfaces. Unfortunately, the unstable interfaces between Mg and the coating layers might be a potential limitation of these coating methods. Therefore, a new strategy that can avoid the drawbacks of conventional approaches such as alloy designs and coatings while decreasing the Mg corrosion rate is highly demanded.

Biomolecules may provide ideal solutions to the above challenges. Biocompatibiltiy and interfacial issues can be avoided since biomolecules such as amino acids and peptides are endogenous materials and spontaneously adsorb onto metal surfaces in aqueous environments^29^. Furthermore, the diverse chemical functionalities of biomolecules may potentially be utilized to modify Mg corrosion chemistry^30^. Several studies systemically revealed that biomolecules affect Mg corrosion under different environment^31–35^. Specifically, Wagener *et al*. reported that proteins in cell culture medium influence the formation of a Ca-P corrosion layer by adsorbing on the Mg surface^34^. Moreover, Lamaka *et al*. investigated the Mg corrosion inhibiting efficiency of various chemical compounds including biomolecules such as acetic acid, glucose, glycoric acid, lactic acid, and malic acid^35^. Proper concentrations of those biomolecules showed substantial effect of suppressing corrosion on pure Mg and some Mg alloys. Such recent reports exhibited the high potential of biomolecules as Mg corrosion inhibitors and the importance of verifying their roles in Mg biodegradation.

**Figure 1:**
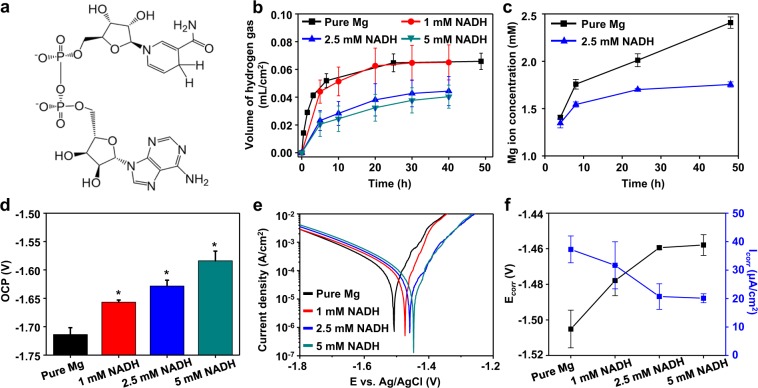
Corrosion behaviors of Mg in HBSSs with different concentrations of NADH at 37nd fa(**a**) Structure of NADH under physiological conditions (pHncentrati(**b**) The amount of hydrogen gas evolved during the corrosion of Mg immersed in HBSSs with different concentrations of NADH (black: 0 mM, red: 1 mM, blue: 2.5 mM, and green: 5 mM). (**c**) Concentrations of Mg^2+^ ions released from Mg immersed in HBSSs for 4, 8, 24 and 48 h (black: pure Mg without NADH and red: pure Mg with 2.5 mM NADH). (**d–f**) OCPs (**d**), representative potentiodynamic polarization curves (**e**), corrosion potentials (E_corr_) and corrosion current densities (I_corr_) (**f**) of Mg immersed in HBSSs with different concentrations of NADH. The results of a statistical analysis based on ANOVA one-way test are shown (*Indicates p < 0.05 for differences in values of samples and untreated control group).

In this work, we adopted nicotinamide adenine dinucleotide (NADH) as a model system to understand the potential effects of biomolecules on the corrosion behavior of Mg. NADH, a naturally occurring cofactor *in vivo*, was self-adsorbed onto metal surfaces *via* its various functional groups such as its phosphate, amide, amine, hydroxyl, and pyridine groups (Fig. 1a), eliminating the abovementioned biocompatibility and interfacial issues in conventional methods. Self-adsorbed NADH was determined by immersion tests and electrochemical and spectroscopic analyses to efficiently decrease the corrosion rate of Mg. Mechanistic investigations showed that NADH efficiently blocked ionic species in a corrosive environment from Mg and further promoted the formation of stable protective layers composed of Ca-P compounds. Finally, based on its proven ability of NADH to inhibit the corrosion of Mg, we confirmed the biocompatibility of NADH through *in vitro* cell study, showing its potential to be clinically utilized as a corrosion inhibitor for Mg-based implantable devices.

## Results and Discussion

### Effect of NADH on Corrosion Behaviors of Mg

The ability of NADH to decrease the Mg corrosion rate was examined using conventional immersion tests, as previously reported^36^. We prepared Hank’s balanced salt solution (HBSS) (8.0 g/L NaCl, 0.4 g/L KCl, 0.14 g/L CaCl_2_, 0.35 g/L NaHCO_3_, 1.0 g/L C_6_H_6_O_6_ (glucose), 0.2 g/L MgSO_4_•7H_2_O, 0.1 g/L KH_2_PO_4_•H_2_O, and 0.06 g/L Na_2_HPO_4_•7H_2_O) with four different concentrations of NADH (ranging from 0 to 5 mM) as immersion solutions. Mg samples were placed in each solution and the amount of hydrogen gas that evolved from each solution, which is related to the Mg corrosion rate, was recorded over time^37^. We found that the amount of evolved hydrogen gradually decreases with increasing concentrations of NADH in solution (Fig. 1b). For example, after 40 h of immersion, the amount of hydrogen gas evolved from pure Mg immersed in HBSS without NADH is 0.066 mL/cm^2^, whereas only 0.040 mL/cm^2^ is evolved from HBSS with 5 mM NADH. In the case of the solutions with 1 or 2.5 mM NADH, the volumes of produced hydrogen gas are 0.065 and 0.044 mL/cm^2^, respectively. Moreover, we directly measured the concentration of Mg^2+^ ions in HBSS to determine the quantity of Mg dissolved during immersion. As shown in Fig. 1c, the Mg^2+^ ion concentrations generated from pure Mg dissolution without NADH and with 2.5 mM NADH are 2.4 and 1.75 mM, respectively, after 48 h of immersion. These results indicate that the dissolved NADH reduces the corrosion rate of Mg.

Electrochemical tests, which are another indicators of the corrosion properties of degradable metals^38^, were performed in four HBSSs with increasing NADH concentrations (0, 1, 2.5, and 5 mM) to further investigate the effects of NADH on Mg corrosion (Fig. 1d). First, open circuit potential (OCP) values increase with NADH concentration in solution, indicating that Mg exhibits nobler behavior in NADH-containing HBSS than in pure HBSS. Second, potentiodynamic polarization curves of Mg immersed in solutions with different NADH concentrations were recorded for measuring corrosion potentials (E_corr_) and corrosion current densities (I_corr_) (Fig. 1e). The I_corr_ values were calculated by using the Tafel extrapolation method^39^. As NADH concentrations of the solutions increase, less negative E_corr_ and lower I_corr_ of Mg are achieved (Fig. 1f). For instance, the I_corr_ of Mg immersed in pure HBSS is 37.3 μA/cm^2^, decreasing to 20.7 μA/cm^2^ when treated with 2.5 mM NADH. Such shifts in the values of E_corr_ and I_corr_ confirm that the corrosion resistance of Mg evaluated by electrochemical analayses also enhances with increasing concentrations of NADH treated.

Interestingly, the corrosion inhibitive effect of NADH was also found in Mg-3wt% Ca alloy, which has a siginificantly higher corrosion rate compared to pure Mg. As shown in Fig. S1, the corrosion rate of the alloys gradually decreased upon the addition of NADH, similar to our observation using pure Mg. This result provides another evidence for the role of NADH as a Mg corrosion inhibitor and also implies its potential usage for other Mg-based alloys.

### Characterization of Corrosion Product from NADH-Treated Mg

**Figure 2:**
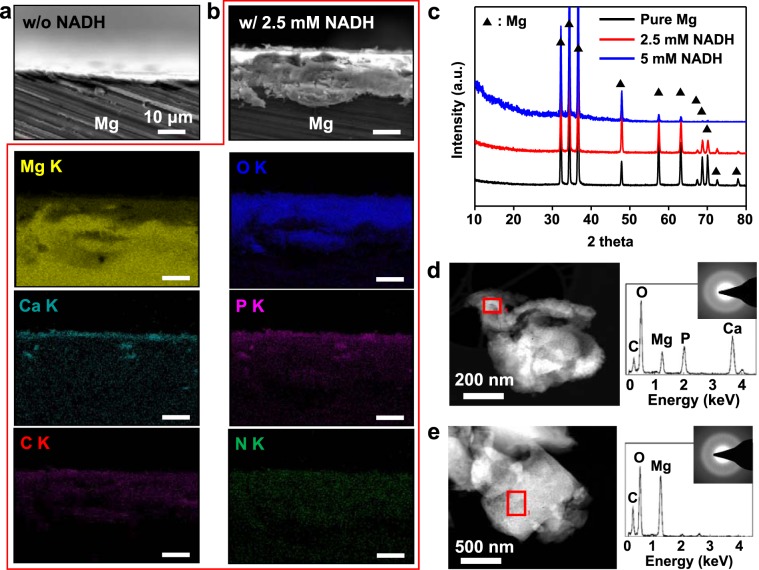
Microscopic analyses for corrosion product layer formed on NADH-treated Mg. (**a**,**b**) Cross-sectional SEM images of pure Mg after immersion in HBSSs without (**a**) and with (**b**) 2.5 mM NADH for 48 h. EDS mapping images for the cross-sectional SEM image of Mg immersed in 2.5 mM NADH-containing HBSS for 48 h are shown in (**b**) with six types of elements (yellow: magnesium, blue: oxygen, cyan: calcium, pink: phosphorous, red: carbon, and green: nitrogen). (**c**) XRD patterns of Mg after 48 h of immersion in HBSSs with different concentrations of NADH (black: 0 mM, red: 2.5 mM, and blue: 5.0 mM). Crystalline Mg peaks are indicated by filled triangles. (**d**,**e**) TEM images, SAED patterns, and TEM-EDS spectra of amorphous Ca-P compounds (**d**) and Mg oxides (**e**) formed on the Mg surface after immersion tests.

To understand the different corrosion behaviors exhibited by Mg upon addition of NADH, we compared surface characteristics of the Mg corroded from 48 h of immersion. Even with the decreased corrosion rates investigated using various methods (Fig. 1b–f), a denser and thicker corrosion product layer is formed as the concentration of NADH increases (Fig. S2). Indeed, a negligible amount of white corrosion product is observed on the surface of Mg in the absence of NADH, while the corrosion product completely covers the Mg surfaces when 5 mM NADH is dissolved in the solution (Fig. S2a–d). The corroded Mg surfaces were analyzed by using scanning electron microscopy (SEM) and energy dispersive X-ray spectroscopy (EDS) to gain further insight into the abovementioned phenomena (Fig. S2e–h). Using lower concentrations of NADH for Mg corrosion results in more Cl atoms being detected on the Mg surface. Cl^−^ ions dissolve Mg corrosion products and Mg oxides to form Mg^2+^ ions^17^. Thus, the corrosion products formed in the absence of NADH might be readily dissolved by the additional Cl^−^ ions, resulting in a thinner corrosion layer. In addition, we observe increased quantities of Ca and P atoms on the Mg surface with increasing concentrations of NADH. This result implies that NADH treatment promotes the formation of Ca and P compounds, which could further prevent Cl^−^ ions from dissolving Mg oxide layers. Cross-sectional SEM images and the corresponding EDS mapping of Mg immersed in 2.5 mM NADH solution for 48 h clearly demonstrate that Ca-P compounds are formed on the surface of Mg oxide layers in the presence of NADH (Fig. 2a,b).

X-ray diffraction (XRD) and transmission electron microscopy (TEM) analyses were performed to obtain detailed information of the corrosion product layer induced by NADH (Fig. 2c–e). No distinct crystalline peaks other than those from Mg are found in the XRD patterns, indicating that components of the layer are amorphous (Fig. 2c). This result matches well with two selected area electron diffraction (SAED) patterns of the layer, both of which exhibit ring patterns typical of amorphous compounds (Fig. 2d,e). TEM-EDS spectra confirm that the amorphous layer comprises Mg, Ca, P, and O atoms. All of these spectroscopic analyses reveal that amorphous Mg oxides and Ca-P compounds are the primary components of the corrosion product layer of Mg and that its production is promoted in the presence of NADH.

### Mechanism of NADH-induced Corrosion Inhibition

**Figure 3:**
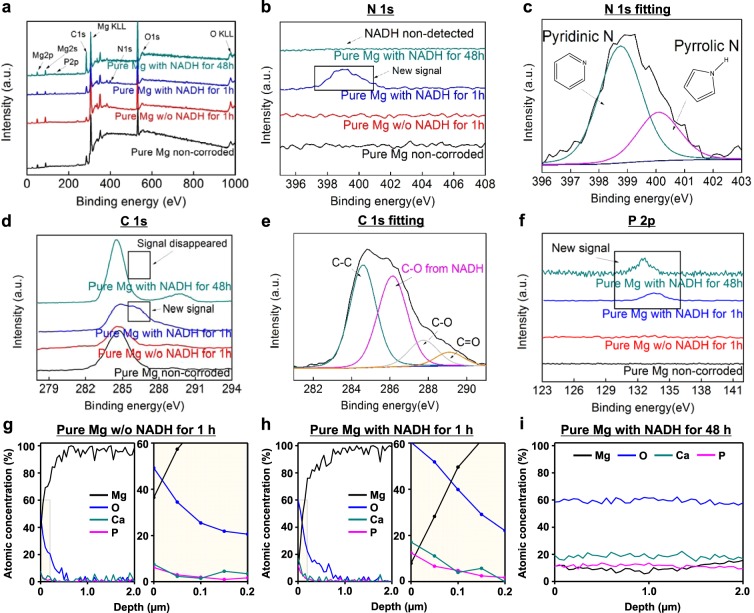
XPS spectra of Mg surface after immersion in HBSSs without and with 2.5 mM NADH: (**a**) the entire range of the binding energy survey for Mg surfaces after immersion in HBSSs without and with NADH for 1 and 48 h (black: Mg before immersion, red: Mg immersed in HBSS without NADH for 1 h, blue: Mg immersed in HBSS with 2.5 mM NADH for 1 h, and cyan: Mg immersed in HBSS with 2.5 mM NADH for 48 h). (**b**) Detailed N 1 s spectra for each spectrum in (**a**). (**c**) Curve fitting of N 1 s spectra for Mg surface immersed in HBSS with NADH for 1 h. Distinct pyridinic and pyrrolic peaks are clearly observed. (**d**) Detailed C 1 s spectra for each spectrum in (**a**). (**e**) Curve fitting of C 1 s spectra for Mg surface immersed in HBSS with NADH for 1 h. C-O peak appears in spectrum of Mg immersed in HBSS with NADH for 1 h, whereas the peak disappeared after 48 h of reaction. (**f**) Detailed P 2p spectra for each spectrum in (**a**). (**g**,**h**) Entire (left) and magnified (right) depth profiles of the corrosion product layer formed on the Mg surfaces after immersion in HBSS without (**g**) and with (**h**) NADH for 1 h. (**i)** Depth profile of the corrosion product layer formed on the Mg surfaces after immersion in HBSS with NADH for 48 h.

To clarify the mechanism of corrosion inhibition induced by NADH, we investigated the chemistry on the Mg surface, where corrosion occurs over time, by using X-ray photoelectron spectroscopy (XPS). Figure 3a depicts the entire range of XPS spectra of Mg surfaces immersed in HBSSs with and without 2.5 mM NADH. Distinct N and P peaks are found after 1 h of reaction in the presence of NADH, whereas those peaks are missing when NADH was not used. The N 1 s spectrum clearly shows that the new N signal that appears after 1 h of NADH treatment can be assigned to pyridinic N (398.8 eV) and pyrrolic N (400.1 eV) atoms from the NADH (Fig. 3b,c). In addition, a new C peak at a binding energy of 286.2 eV, which can be assigned to C-O bonds in NADH, is found at the Mg surface after 1 h of reaction in the presence of NADH (Fig. 3d,e). Since negatively charged biomolecules adsorb onto the surfaces of metal oxides via hydrogen bonds or electrostatic interactions^29^, we believe that NADH adsorbs onto the Mg oxides during the initial stage of corrosion. However, the XPS spectra generated in the presence of NADH change significantly after 48 h of reaction. N and C peaks that appeared after 1 h of reaction, assigned to self-adsorbed NADH, disappear after 48 h (Fig. 3b,d). Changes in these peaks indicate that NADH is no longer present in the outermost layer of the Mg in the late stages of corrosion. Instead, the outermost surfaces are covered primarily with amorphous Ca-P compounds and Mg oxides, as shown in the abovementioned SEM and TEM results (Fig. 2). The P peaks do not disappear, even after 48 h of reaction, as they originate from Ca-P compounds formed on the corroded Mg surface (Fig. 3f).

For further demonstrating chemical nature of the corrosion product layer formed by NADH, we also obtained the XPS depth profiles of the layers formed on the Mg surfaces immersed in HBSSs with and without 2.5 mM NADH. Substantially high compositional concentrations of Ca and P atoms were observed in the outmost layer formed on NADH-treated Mg (Ca: 17.2% and P: 12.5%) after 1 h of corrosion, compared to those formed on non-treated Mg (Ca: 7.6% and P: 6.2%) (Fig. 3g,h). This result indicates that the self-adsorbed NADH onto the Mg surface at the early stage of corrosion may initiate and promote formation of Ca-P compounds. After 48 h of immersion with NADH, we found that the layer with high atomic concentration of Ca and P atoms (average value, Ca: 18.5% and P: 11.5%) were broadened, indicating a formation of thick, protective layer. Additionally, consistent with our TEM results for corrosion product layer (Fig. 2), Ca, P, Mg, and O peaks, which could be assigned to Ca-P compounds and Mg oxides, were continuously observed until 2 µm depth (Fig. 3i).

**Figure 4:**
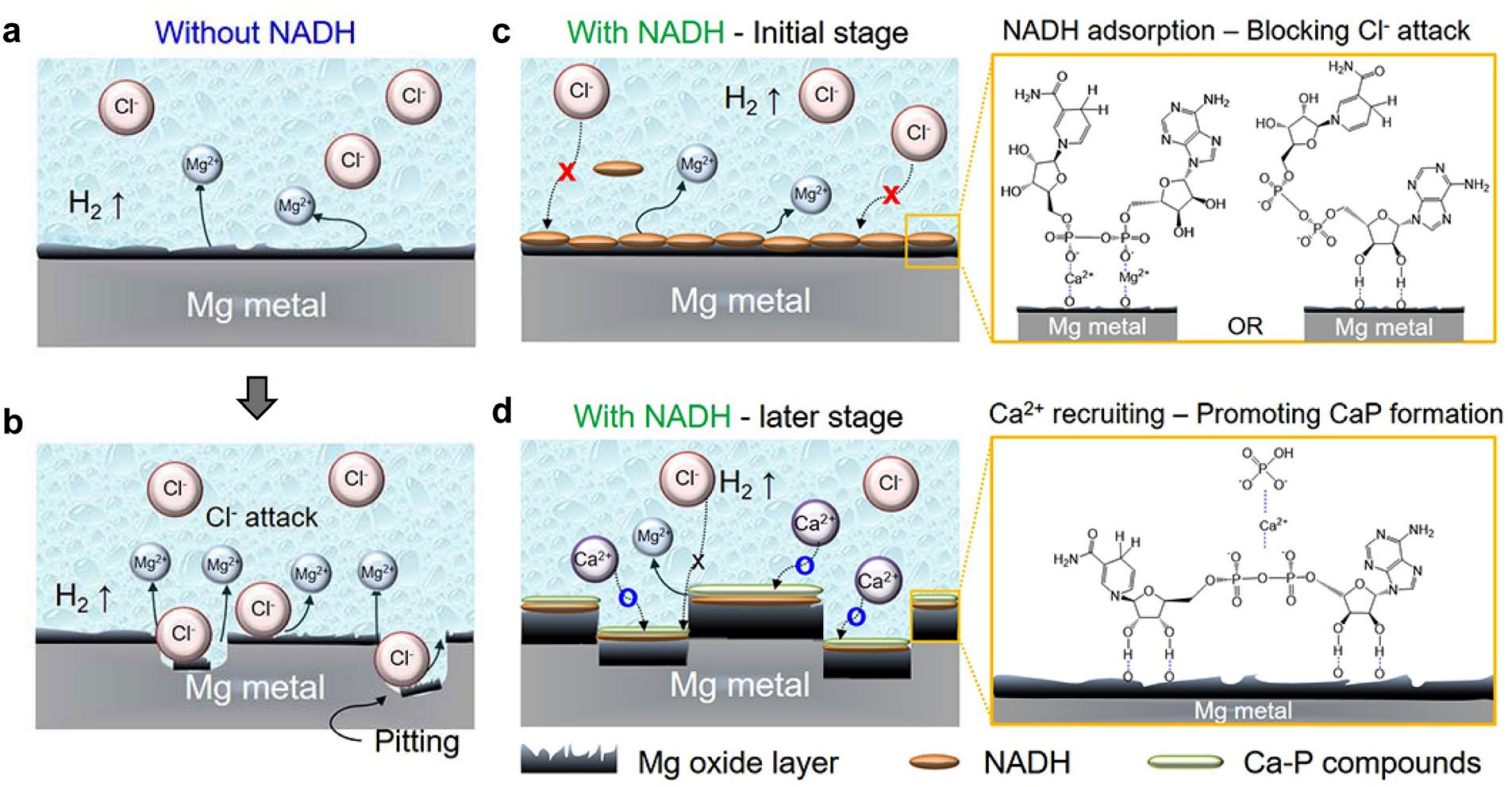
Schematic illustrations of the role of NADH in the corrosion of Mg: (**a**,**b**) conventional corrosion of Mg in the absence of NADH. Here, Cl^−^ ions attack and dissolve Mg oxides formed on the Mg surface. (**c**,**d**) Corrosion of Mg in the presence of NADH. At the initial stage of corrosion (**c**), NADH self-adsorbs onto Mg oxides and blocks Clf ions from dissolving Mg oxides. At the late stages of corrosion (**d**), self-adsorbed NADH recruits Ca^2+^ ions to form stable Ca-P compounds on Mg oxides.

Based on the analyses for the chemical nature of the Mg surface and the corrosion product layer using XPS, we propose a possible mechanism of NADH-induced corrosion inhibition during the initial and late stages of the corrosion process (Fig. 4). We believe that self-adsorbed NADH might play a crucial role in reducing the Mg corrosion rate during the initial stages. Typically, Cl^−^ ions cause Mg corrosion products such as Mg(OH)_2_ to dissolve and form Mg^2+^ ions via the following reaction:

1 $${\mathrm{Mg}(\mathrm{OH})}_{{\rm{2}}}+2{{\rm{C1}}}^{-}\to {\rm{MgC}}{1}_{2}({\rm{aq}})+2{{\rm{OH}}}^{-}$$

Due to such reactions with Cl^−^ ions, Mg surfaces are continuously exposed to the solution. Thus, corrosion proceeds steadily because protection layer is not formed (Fig. 4a,b). In the presence of NADH, however, NADH self-adsorbed on the Mg oxide layers prevents Cl^−^ ions from dissolving Mg oxides via the following equation:

2 $${\rm{Mg}}{({\rm{OH}})}_{2}+{\rm{NADH}}\to {\rm{Mg}}{({\rm{OH}})}_{2}\ldots {\rm{NADH}}$$

Thus, one of the ways that NADH inhibits Mg corrosion is by blocking Cl^−^ ion attacks and promoting the formation of thick corrosion products on the Mg surface that function as protection layers. (Fig. 4c). A role of NADH during late stages of corrosion might be to promote the formation of Ca-P compounds. At physiological pH, the negative charges of self-adsorbed NADH could attract and accumulate nearby Ca^2+^ ions to its phosphate groups (Fig. 4d). The increased local concentration of Ca^2+^ ions might cause Ca-P compounds to be formed by reacting with H_2_PO_4_^−^ or HPO_4_^2−^ ions, as described by following equation^23^:

3 $${\rm{Mg}}{({\rm{OH}})}_{2}\cdots {\rm{NADH}}+{{\rm{Ca}}}^{2+}+{{\rm{H}}}_{2}{{{\rm{PO}}}_{4}}^{-}\,{\rm{or}}\,{{{\rm{HPO}}}_{4}}^{2-}\to {\rm{Mg}}{({\rm{OH}})}_{2}\cdots {\rm{NADH}}\cdots {\rm{Ca}} \mbox{-} {\rm{P}}\,{\rm{compounds}}$$

The newly formed Ca-P compounds might also contribute to protecting from Cl^−^ attacks since they are more stable than Mg oxides in the presence of Cl^−^ ions^40^. We believe that NADH has bi-functional effects to reduce Mg corrosion. One mechanism involves blocking Cl^−^ ions by self-adsorbing onto Mg oxides at the early stage of corrosion. The other is the recruitment of Ca^2+^ ions, which can form stable Ca-P compounds on the outermost surfaces during the late stages of corrosion.

### Feasibility of NADH as a Corrosion Inhibitor for Mg-Based Implantable Devices

**Figure 5:**
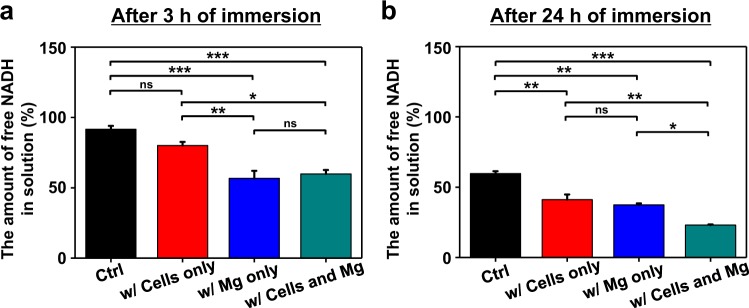
Quantification of NADH incorporated in cell culture medium. The amount of NADH was measured in four different groups: only 1 mM NADH-dissolved medium (black, Ctrl), 1 mM NADH-dissolved medium with L929 cells (red, w/Cells only), 1 mM NADH-dissolved medium with Mg specimens (blue, w/Mg only), and 1 mM NADH-dissolved medium with both L929 cells and Mg specimens (cyan, w/Cells and Mg). (**a**,**b**) The amount of free NADH concentration in solution after 3 h (**a**) and for 24 h (**b**) of immersion. Statistical significance was described as *(p < 0.05), **(p < 0.01), and ***(p < 0.001).

To understand the behavior of NADH in physiological environments, we quantitatively monitored changes in the concentration of NADH under four different conditions: only 1 mM NADH-dissolved medium (group 1), 1 mM NADH-dissolved medium with L929 cells (group 2), 1 mM NADH-dissolved medium with Mg specimens (group 3), and 1 mM NADH-dissolved medium with both L929 cells and Mg specimens (group 4) (Fig. 5). At the initial stage of corrosion process (after 3 h of immersion), we found that the amount of NADH remained in group 3 is noticeably lower than those of group 1 and group 2. Although it is difficult to accurately calculate the amount of NADH adsorbed onto Mg surface based on free NADH concentration, we believe that some portion of NADH in the solution could be adsorbed onto Mg surfaces at the initial stage, leading to a decrease in the concentration of free NADH in group 3. Moreover, there was no significant difference between group 3 and group 4 (1 mM NADH medium with Mg and L929 cells), supporting that decrease in free NADH concentration might be related to its adsorption process onto Mg surface. The small changes in the NADH concentration in group 1 and group 2 were probably originated from NADH degradation in phosphate-containing buffer or cell metabolism process^41–45^.

Interestingly, at the later stage of corrosion process (after 24 h of immersion), free NADH concentrations in group 2 and group 4 become statistically lower than those in group 1 and group 3, respectively (Fig. 5b). These results might be originated from increased amount of NADH used by proliferated cells in the medium for 24 h. However, it should be noted that difference in free NADH concentration depending on the presence of Mg has remained even after 24 h of immersion. As shown in Fig. 5b, free NADH concentrations in Mg-containing groups (group 3 and group 4) were significantly lower than those in other groups (group 1 and group 2), respectively, regardless of the presence or absence of cells. Put together, we believe that these NADH quantification results showed that NADH could be adsorbed onto Mg surface before being consumed by cells *in vitro*.

To further demonstrate the feasibility of NADH as a corrosion inhibitor of Mg *in vivo*, we investigated the stability of NADH-induced protective layer formed onto the Mg surface under the vigorously stirred medium, which mimic dynamic fluid flow under physiological condition. The same microscopic and spectroscopic analyses described in Figs 2b and 3i were performed using Mg specimen immersed in 2.5 mM NADH solution stirred with a speed of 100 rpm (Fig. S3). When compared both the cross-sectioned SEM images and the XPS depth profiles shown in Fig. S3 and Figs 2b and 3i, the thickness, density, and the chemical nature of the protective layer formed under the static and dynamic conditions show no significant difference between each other. Such results imply that NADH could also functions well as a promoter of protective layer formation by being adsorbed onto the Mg surfaces even under the dynamic physiological environment.

**Figure 6:**
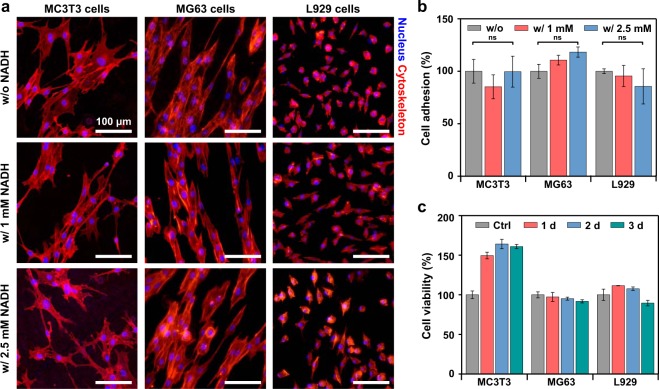
Cytotoxicity of osteoblastic (MC3T3 and MG63) and fibroblastic (L929) cells cultured with Mg without and with NADH treatment. (**a**) Confocal microscopy images of MC3T3, MG63, and L929 cells adhered to the surface of non-treated and 1 and 2.5 mM NADH-treated Mg (blue: nuclei of cells and red: cytoskeleton and). (**b**) The relative number of MC3T3, MG63, and L929 cells per cm^2^ adhered to the non-treated (gray) and 1 (red) and 2.5 (blue) mM NADH-treated Mg surface. According to statistical analysis based on ANOVA one-way test, the means of each group show no difference (ns: not significant). (**c**) Viability of MC3T3, MG63, and L929 cells cultured with Mg treated with 2.5 mM NADH, compared to those cultured only in the medium (gray: Ctrl).

Moreover, the cytotoxicity tests were performed to investigate biocompatibility of NADH treatment. Osteoblastic cells (MC3T3 and MG63) and fibroblastic cells (L929) were chosen for evaluating the biological effects of NADH as biodegradable Mg alloys are generally used for bone-related implants in clinical settings. The quantities, adhesion characteristics, and morphologies of the three types of cells attached to 1 and 2.5 mM NADH-treated Mg surface showed no difference, compared to those attached to non-treated Mg (Fig. 6a,b). Moreover, viability of the cells was analyzed using a CCK-8 assay (Fig. 6c and Fig. S4). All three cells cultured with 2.5 mM NADH-treated Mg represent over 90% of the viability upon 3 days compared to the control, confirming negligible cytotoxicity. These results demonstrate that NADH treatment does not adversely affect the viability and functions of cells, indicating that our approach has potential to be utilized *in vivo* with its excellent biocompatibility.

In addition, a wide range of delivery method is applicable for NADH due to its self-adsorbing nature. As discussed, NADH can be directly adsorbed onto the Mg surfaces just by immersing Mg in NADH solutions within a few hours. Therefore, we think that dropping or spraying NADH solutions near Mg after implanted for delivering NADH to the Mg surfaces can be a possible approach for directly introducing NADH to Mg implants. However, direct delivery of NADH to Mg implantation sites could result in fast degradation of NADH as consumed by tissues or diluted by bio-fluids. Moreover, certain clinical conditions require slow and extended release of corrosion inhibitors over time. Therefore, integration of polymer materials with NADH could provide a promising way to prevent NADH from quickly being consumed by tissues or being diluted by bio-fluids after implantation. We demonstrated the possibility of this strategy by using PLLA as a NADH carrying material (Fig. S5). Such approach of embedding NADH in conventional polymer matrix shows the alternative application of NADH, which could be further optimized in various ways for usage in clinical settings.

## Conclusion

We devised a novel method of using NADH, which is a typical cofactor *in vivo*, to improve the corrosion resistance of Mg for biomedical implantable system. Characterizations of corrosion behaviors revealed that Mg corrosion rates are exceptionally suppressed when NADH is dissolved in the reaction solution; this property was confirmed by combined microscopic and spectroscopic analyses to be caused by NADH-promoted formation of protective layers. The mechanism by which NADH is active in Mg biodegradation was investigated and NADH was found to have two roles: (1) self-adsorption onto Mg oxides to prevent Cl^−^ ions from dissolving Mg oxides in the initial stage and (2) recruitment of local Ca^2+^ ions to promote the formation of stable Ca-P compounds in the late stages. Furthermore, we demonstrated that NADH stably functions as a corrosion inhibitor of Mg under physiological environment. Moreover, excellent *in vitro* biocompatibility of NADH showed high potential to be applicable for *in vivo* study. Owing to its simplicity and novelty, we believe our NADH-utilizing strategy can be further extended to various biodegradable metals and assist in developing an understanding of the roles of biomolecules in metal biodegradation.

## Materials and Methods

### Materials preparation

Pure Mg (99.98 wt%) plates were obtained from YinGuang Magnesium Industry. Mg-3wt%Ca alloy was fabricated by casting process using pure Mg (99.98 wt%) and pure Ca billet (99.99 wt%, RNDKorea). Disks with diameters and thicknesses of 8 and 1 mm, respectively, were ground using a series of SiC grinding papers, ending at 2000 grit. All disks were cleaned ultrasonically in ethanol and acetone, and then finally dried in flowing air. NADH (≥97%, HPLC, Sigma-Aldrich) and PLLA (poly(L-lactide), ester-terminated, Sigma-Aldrich) were used without further purification. To prepare NADH-embedded PLLA coated Mg specimens, 447 mg of PLLA was first dissolved in 10 mL of chloroform to yield a concentration of 3 wt%. Then, 17.5 mg of NADH powder was poured into this solution. Ultrasonication, rotation, and vortexing were used to agitate and disperse NADH in the PLLA solution. Finally, 200 μL of the prepared solution was dropped onto each side of Mg specimen and fully dried for 1 h under ambient conditions, thereby coating 1 μmol of NADH-embedded PLLA.

### Immersion test

Immersion tests were performed in HBSS at 37 ± 0.5 °C. NADH powder was added to HBSS, and the mixture was stirred vigorously using a stirring bar for at least 30 min prior to immersion testing. Disk-shaped Mg samples were suspended in the solution described above and funnels were placed over the specimens to collect the hydrogen gas that evolved from them. Thus, the volume of hydrogen produced was measured over time. As a control, the amount of hydrogen gas evolved from a specimen submerged in pure HBSS was measured.

### Electrochemical tests and Mg^2+^ Ion assay

Electrochemical tests were performed using an electrochemical cell with a conventional three-electrode system and a potentiostat (CHI 760C, CH Instruments, Inc.) at 37 ± 0.5 °C. An Ag/AgCl (BASi, 3 M NaCl) electrode was used as the reference electrode, and a platinum plate was used as the counter electrode. OCP tests were performed for 1800 s immediately after the samples were immersed in the solution. To obtain potentiodynamic polarization curves, Mg specimens with an exposed surface area of 0.25 cm^2^ were used as the working electrode and tested at a scan rate of 1 mV/s from −2 V to −1 V. The tests were performed after 30 min of immersion of the samples in the solution, in order to observe the corrosion resistance of Mg after NADH was adsorbed onto the surfaces^39^. All of the electrochemical tests were performed in HBSSs without and with NADH (1, 2.5, and 5 mM). We used Mg ion assay kits (QuantiChrom^TM^ Magnesium Assay Kit, BioAssay Systems) at specific times after Mg sample immersion to measure the amount of Mg^2+^ released.

### Materials characterization

XPS analysis was performed using a PHI 5000 instrument from VersaProbe. Monochromated Al Κα X-rays were used for measuring approximately the first 10 nm of each sample under a vacuum of 2.0 × 10^−7^ Pa. A spot with a radius of 100 μm was used. For acquiring XPS depth profiles, corrosion product layer formed on the Mg surfaces was sputtered at a rate of 50 nm/min. The top-view and cross-sectional morphologies of the samples were investigated via SEM (Inspect F50, FEI Co., USA) after immersion testing, and their chemical compositions were studied using EDS (Apollo XL, AMETEK Co., USA). The phases of the corroded surfaces were analyzed using XRD (D/MAX-2500/PC, Rigaku Co., Japan) with a Cu target over a 2θ range from 10° to 90°. The step size was 0.01°. TEM analysis was performed using an FEI Titan 80–300 microscope. For TEM sample preparation, corroded Mg layers were immersed in 5 mM HBSS with NADH for 48 h, carefully scraped off, and suspended in ethanol. Then, 20 μL of the above ethanol solution was dropped onto a TEM grid placed on filter paper, which was then dried at 80 °C for 2 h.

### Cell cytotoxicity

To evaluate cytotoxicity of NADH treatment on Mg, MC3T3, L929, and MG-63 cells were cultured in Dulbecco’s modified Eagle’s medium (DMEM) with 10% fetal bovine serum (FBS) and 1% penicillin-streptomycin (GIMCO™) in a humidified atmosphere with 5% CO_2_ at 37 °C. Mg specimens were immersed in different concentrations of NADH-dissolved cell culture medium for 48 h. After the NADH treatment, the Mg specimens were incorporated into the medium where the cells were immediately seeded at a density of 7 × 10^3^ cells/sample. The number of cells adhered to the Mg surface and cellular morphologies were investigated using a confocal laser scanning microscopy (LSM 700, Carl Zeiss) after culturing for 48 h. The cytoskeletal structure and nuclei were stained with rhodamine-phalloidin and 4',6-diamidino-2-phenylindole (DAPI), respectively. Fixation of cells was also performed by applying 4% paraformaldehyde for 20 min, and permeabilization was performed with a cytoskeleton buffer for 10 min. After this step, Mg samples were blocked via incubation with 5% FBS and 0.1% Tween-20 in phosphate-buffered saline (PBS) solution. Rhodamine-phalloidin dyes in a blocking buffer were added, and the samples were incubated for 90 min as a final step. To investigate cell viability, the cells were seeded in a 96-well culture plate at a density of 5 × 10^3^ cells/well and incubated for 24 h with pure Mg and 2.5 mM NADH-treated Mg, respectively. After incubation, CCK-8 assay was used for the determination of cell viability, which was measured at 410 nm using a microplate reader (Glomax Discover System, Promega). The cells incubated without any Mg sample were set to have a viability of 100%, and the relative values were calculated.

### Quantification of NADH

To investigate the effectiveness of NADH treatment on Mg under physiological environment, the amount of NADH remained in the solution containing Mg and cells was determined using NADH quantification kit (Sigma-Aldrich, USA). L929 cells were used for the tests. A 10 µmol of NADH was dissolved in 10 mL of each culture medium to generate a 1 mM NADH solution. The amount of NADH in the solution incorporated without anything (control), with 7 Mg specimens only, with cells at a density of 1.44 × 10^6^ cells/ml only, and with both 7 Mg specimens and cells at a density of 1.44 × 10^6^ cells/ml were measured over 48 h. At each time point, 1 µL of each incubated solution at 37 °C was collected, diluted and then mixed with assay buffer according to the manufacturer’s instructions. Absorbance at 450 nm of the prepared solution was measured using a microplate reader (Glomax Discover System, Promega). For NADH-release profile analysis of NADH-embedded PLLA coated Mg, same procedures were performed with a 8 mL of HBSS incorporated with 4 specimens at 37 °C.

## Electronic supplementary material

Supplementary Information
