# Supplementary material for: A new corrosion-inhibiting strategy for biodegradable magnesium: reduced nicotinamide adenine dinucleotide (NADH)
Source: Sci Rep. 2018 Dec 10;8:17743. doi: 10.1038/s41598-018-36240-3 (PMC6288270; doi:10.1038/s41598-018-36240-3)
Supplement: Supplementary file 1 — Supplementary Information [file 41598_2018_36240_MOESM1_ESM.docx]

Supplementary Information

A new corrosion-inhibiting strategy for biodegradable magnesium: reduced nicotinamide adenine dinucleotide (NADH)

Jimin Park^‡^, Minjung Park^‡^, Hyunseon Seo^‡^, Hyung-Seop Han, Ji-Young Lee, Dongkyu Koo, Kyeongsoo Kim, Pil-Ryung Cha, James Edwards, Young-Woon Kim, Kang-Sik Lee, Myoung-Ryul Ok, Hojeong Jeon, Hyun-Kwang Seok^*^ & Yu-Chan Kim^*^

**
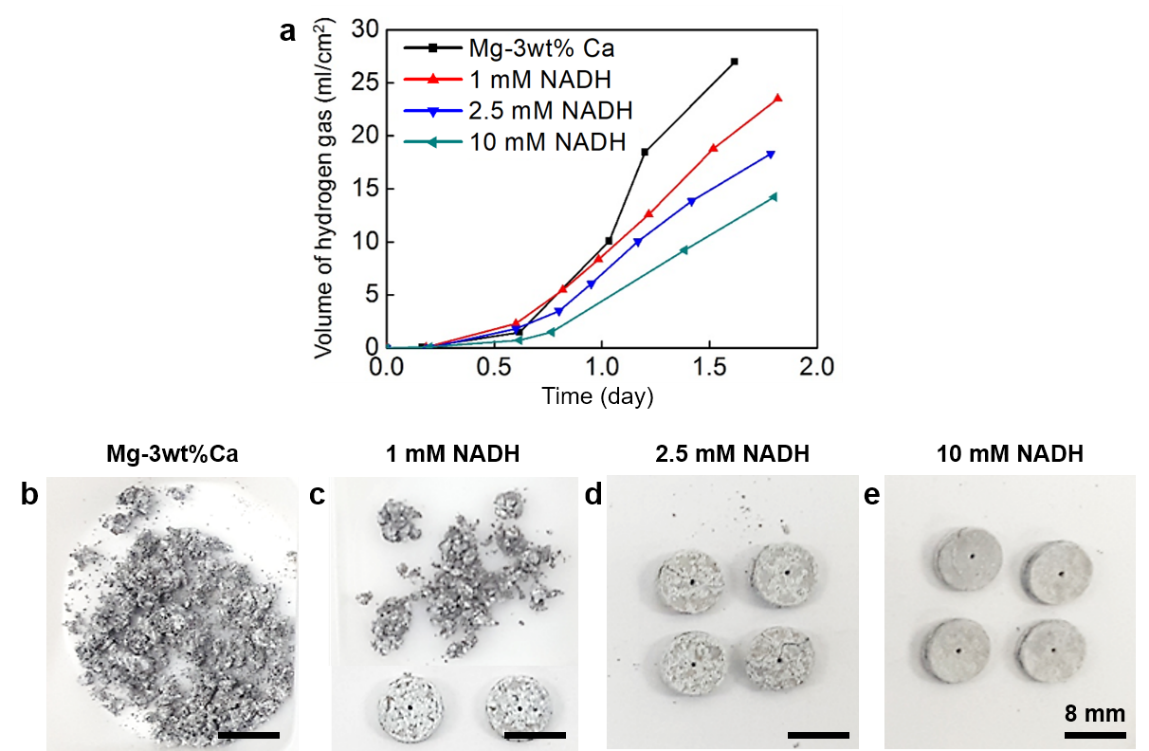
**

**Figure S1.** **a** The amount of hydrogen gas evolved during the corrosion of Mg-3wt%Ca immersed in HBSSs with different concentrations of NADH (black: 0 mM, red: 1 mM, blue: 2.5 mM, and green: 10 mM). **b-e** Optical images of Mg-3wt%Ca specimens after immersion tests (**b**: 0 mM, **c**: 1 mM, **d**: 2.5 mM, and **e**: 10 mM). The greater part of the specimen immersed in HBSS without NADH crumbled due to fast corrosion of Mg-3wt%Ca alloy, while the specimen immersed in 10 mM NADH solution nearly maintained its original shape.


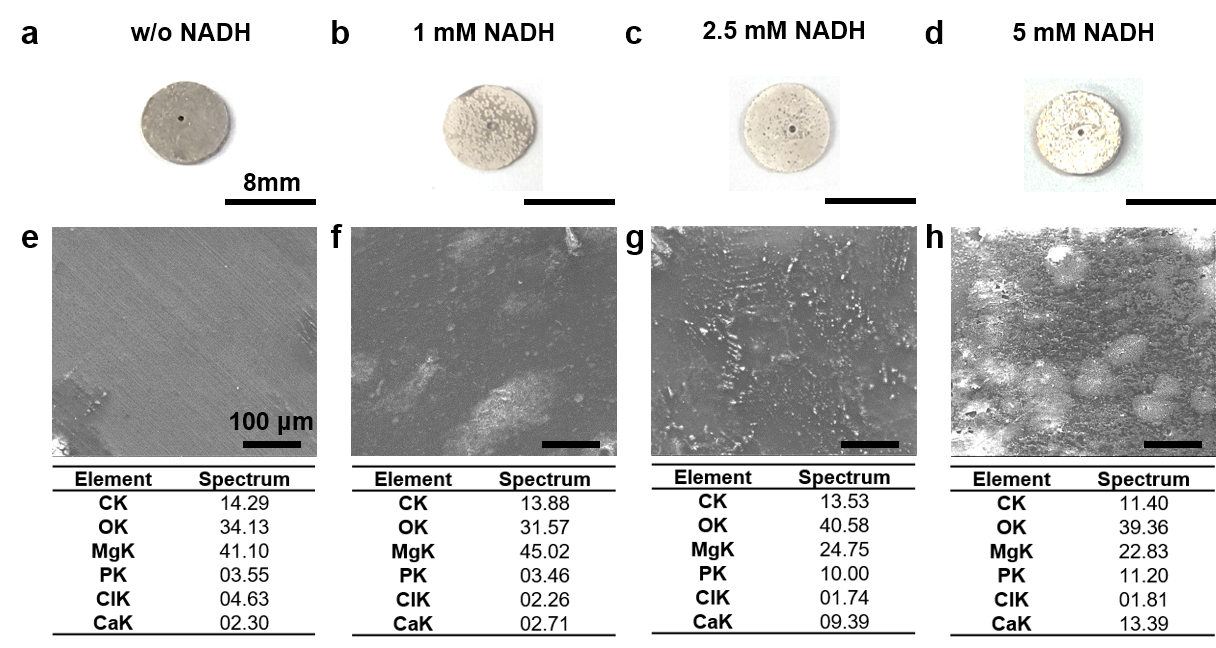


**Figure S2.** Surface characteristics of Mg after immersion test in HBSS with different concentrations of NADH (0 to 5 mM) for 48 h. **a-d** Optical images of Mg specimens after immersion tests. **e-h** SEM images and corresponding EDS data for the surfaces of Mg after immersion tests in HBSSs with different concentrations of NADH (**e**: 0 mM, **f**: 1 mM, **g**: 2.5 mM, and **h**: 5.0 mM).


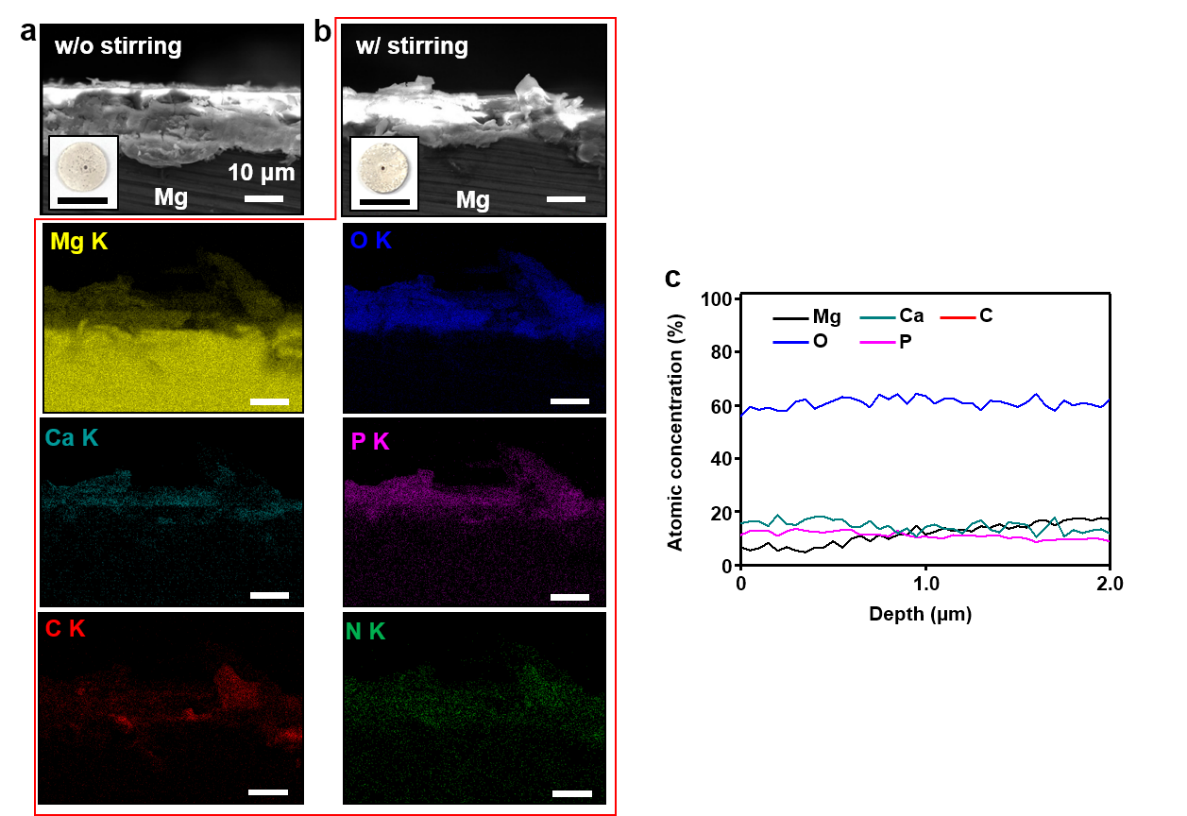


**Figure S3.** Characterization of the corrosion product layer formed on the surface of Mg immersed in the stirred 2.5 mM NADH-containing HBSS (a speed of 100 rpm) for 48 h. **a, b** Cross-sectional SEM images of pure Mg after immersion in 2.5 mM NADH-containing HBSS without (**a**) and with (**b**) stirring for 48 h. EDS mapping images for the cross-sectional SEM image of Mg immersed in the stirred 2.5 mM NADH-containing HBSS for 48 h are shown in (**b**) with six types of elements (yellow: magnesium, blue: oxygen, cyan: calcium, pink: phosphorous, red: carbon, and green: nitrogen). **c** XPS depth profile of the corrosion product layer formed on the Mg surfaces after immersion in the stirred 2.5 mM NADH-containing HBSS for 48 h. Both the thickness and atomic compositions of the layer show no significant difference compared to those of the layer formed under a static condition shown in Figure 3i.


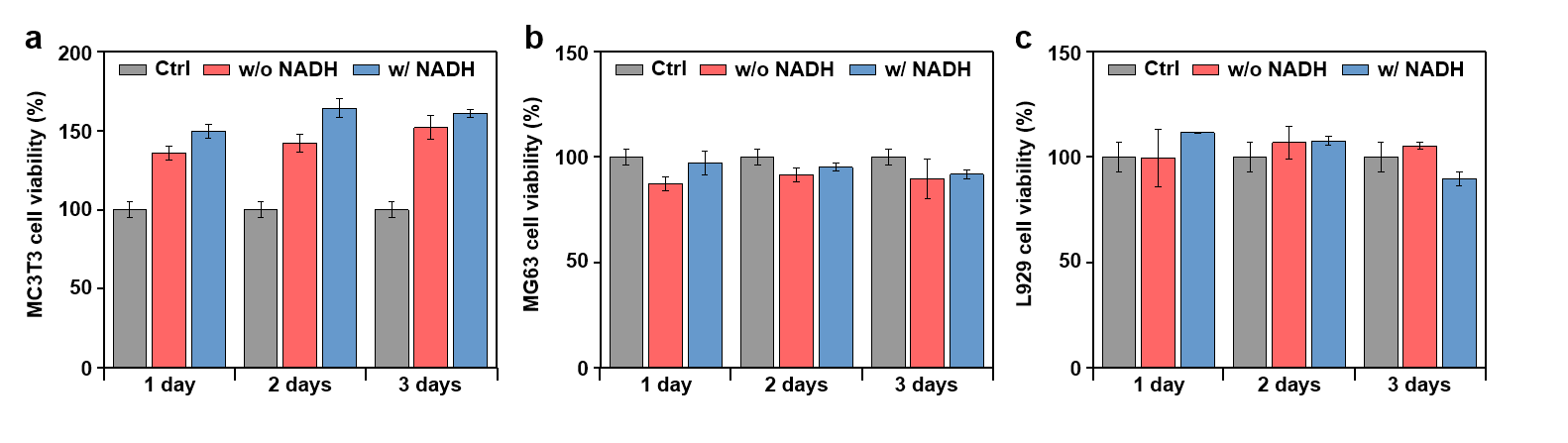


**Figure S4**. Viability of MC3T3 (**a**), MG63 (**b**), and L929 (**c**) cells cultured with Mg treated without NADH (red) and with 2.5 mM NADH (blue), compared to those cultured only in the medium (black: Ctrl). Every group of each cell shows more than 90% of viability, indicating no cytotoxicity.


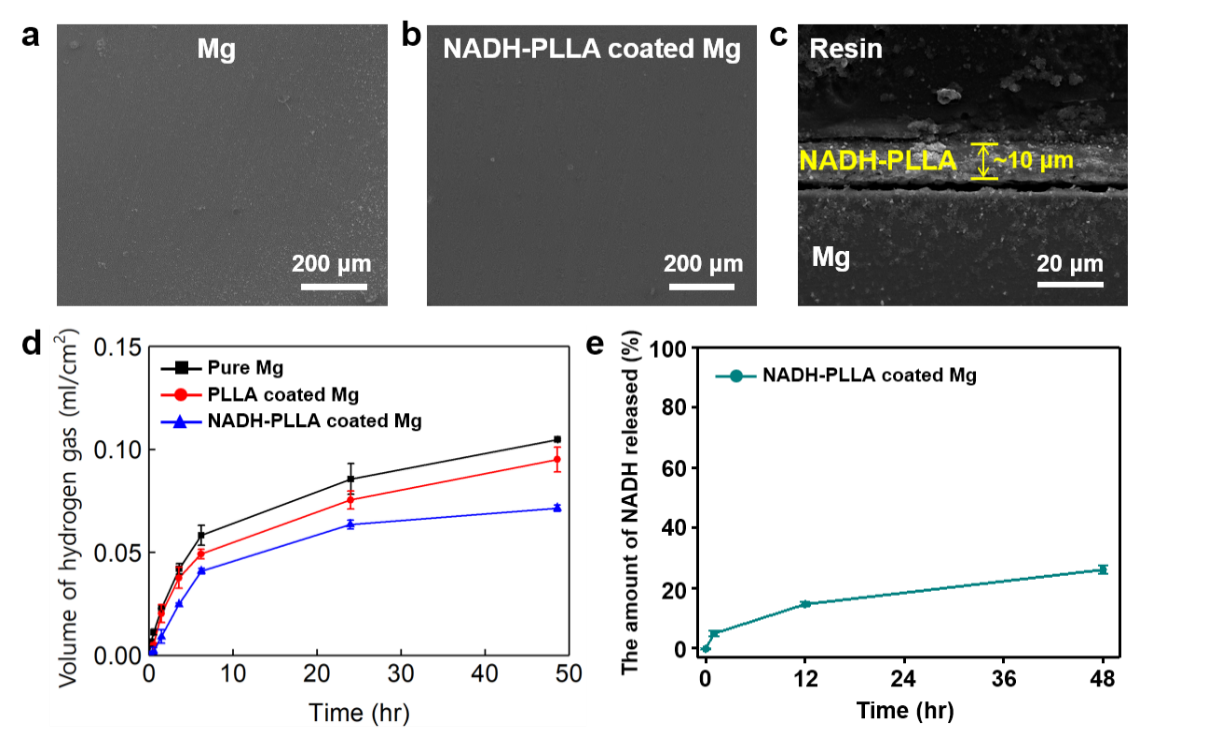


**Figure S5.** **a-c** SEM images of the surface of pure Mg (**a**), and the surface (**b**) and the cross-section (**c**) of NADH-embedded PLLA coated Mg. d The amount of hydrogen gas evolved from pure Mg (black), PLLA coated Mg (red), and NADH-embedded PLLA coated Mg (blue) immersed in HBSSs over 48 h. **d** NADH-embedded PLLA coated Mg showed significantly reduced hydrogen evolution compared to that from pure Mg or PLLA coated Mg. **e** Relative amount of NADH released from NADH-PLLA coated Mg over 48 h. It should be noted that only 5% of total amount of NADH embedded in the PLLA was released from the matrix after 1 h of immersion.
